# A report on COVID-19 epidemic in Pakistan using SEIR fractional model

**DOI:** 10.1038/s41598-020-79405-9

**Published:** 2020-12-17

**Authors:** Zubair Ahmad, Muhammad Arif, Farhad Ali, Ilyas Khan, Kottakkaran Sooppy Nisar

**Affiliations:** 1 Department of Mathematics, City University of Science and Information Technology, Khyber Pakhtunkhwa, Peshawar, 25000 Pakistan; 2 Computational Analysis Research Group, Ton Duc Thang University, Ho Chi Minh City, 70000 Vietnam; 3 Faculty of Mathematics and Statistics, Ton Duc Thang University, Ho Chi Minh City, 70000 Vietnam; 4 Department of Mathematics, College of Science Al-Zulfi, Majmaah University, Al-Majmaah, 11952 Saudi Arabia; 5 Department of Mathematics, College of Arts and Science, Prince Sattam Bin Abdulaziz University, Wadi Al-Dawaser, 11991 Saudi Arabia

**Keywords:** Computational models, Computational biology and bioinformatics

## Abstract

Recently, novel coronavirus is a serious global issue and having a negative impact on the economy of the whole world. Like other countries, it also effected the economy and people of Pakistan. According to the publicly reported data, the first case of novel corona virus in Pakistan was reported on 27th February 2020. The aim of the present study is to describe the mathematical model and dynamics of COVID-19 in Pakistan. To investigate the spread of coronavirus in Pakistan, we develop the SEIR time fractional model with newly, developed fractional operator of Atangana–Baleanu. We present briefly the analysis of the given model and discuss its applications using world health organization (WHO) reported data for Pakistan. We consider the available infection cases from 19th March 2020, till 31st March 2020 and accordingly, various parameters are fitted or estimated. It is worth noting that we have calculated the basic reproduction number ${\mathfrak{R}}_{0} \approx 2.30748$ which shows that virus is spreading rapidly. Furthermore, stability analysis of the model at disease free equilibrium DFE and endemic equilibriums EE is performed to observe the dynamics and transmission of the model. Finally, the AB fractional model is solved numerically. To show the effect of the various embedded parameters like fractional parameter $\alpha$ on the model, various graphs are plotted. It is worth noting that the base of our investigation, we have predicted the spread of disease for next 200 days.

## Introduction

From the beginning the world is constantly facing disasters, in the form of earth quakes, tsunami, floods and pandemics occurred in different times and places. For example in different times the human being suffered by different kinds of infectious diseases, like HIV, Ebola virus, bird flu, dengue virus, malaria, TB, hepatitis B and C, diarrhea, influenza, chicken pox and rubella disease etc. These infectious diseases affected humans as well as other animals. Beside this, these infectious diseases not only spread in human and animals but it also spread in plants like, Pine wilt disease, Moko diseases, sugar cane, orange rust, Karnal bunt diseases and Dutchelm etc.^1^. These pandemics not only affected the economy of the world but also caused many deaths of mankind. The study of these infectious diseases is very important to investigate the spread of infectious diseases and to find out the behavior of these infectious diseases on living organisms. To look for the possible available resources which help to detect these diseases and prevent from them. The duty of humankind is to create a healthy environment in which they live. The growth in human population is increasing with a great speed which also effect the environment. The nature of spreading of these viruses are different, some of them are fast spreading viruses while others are slow in spreading. In-spite of the spreading nature of these infectious diseases, they affected many humans as well as plants.

As discussed above, viruses may have different nature and having some specific visible history for different stages of spreading these infectious diseases. In the present study we formulate the model for the globally spreading infectious disease namely novel corona virus (COVID-19). The first case of COVID-19 in human was reported in Wuhan China on December 31st 2019. Initially, the symptoms of this virus was considered as pneumonia. After the vaccination and treatment of pneumonia the infected patient was not recovered and the treatment was not effective^2^. Secondly, it was observed that the transmission of this virus was very fast from human to human in China. Furthermore, the infected cases were not limited to the city of China Wuhan, it also spread in other cities of China^3^. As the presence of this virus was not limited to China, but also spread in different region of the world. It can be noticed from the behavior of corona virus that initially; it was considered as epidemic disease. But the cases of corona virus spread in the whole world and then it became pandemic disease. Furthermore, it was found that symptoms of corona virus in human being takes 2–10 days^4^.

As this deadly virus spread rapidly in China and in the whole world, like Europe, North America, Germany and Italy and caused many deaths. The number of deaths due to COVID-19 was record in thousands. This virus also affects Asian countries specially Pakistan. In Pakistan the first case of Corona virus was detected in 27th February 2020^5^. From the 27th February 2020 till to 31st March 2020, the number of cases of COVID-19 increased day by day. According to the data collected by WHO^5^ the number of total infected cases were 1865 and total reported deaths were 25 till 31st March 2020.

Fractional order modeling is quite important to understand the realistic situations of the dynamical models. Different researchers use different fractional operators to fractionalize their models. Like, Zhang^6^ modeled the dynamics of COVID-19 in five different compartments and extended the model to the fractional order differentiation to capture different memories using fractional operators of singular and non-singular kernels. Shaikh et al*.*^7^ investigated the spread of the deadly virus COVID-19 in India and fractionalized the classical model with Caputo–Fabrizio (CF) fractional operator. Baleanu et al*.*^8^ reported the numerical solutions of CF time fractional model of COVID-19 with different compartments by using homotopy analysis transform method. Yadav and Verma^9^ examined the spread of COVID-19 in Wuhan, China through a dynamical CF fractional model with different classes and shown the effect of concentration of virus in the surrounding environment on these classes. Lu et al*.*^10^ investigated the dynamics of COVID-19 in different high concentrated cities of China using Caputo fractional model with six different compartments. Rajagopal et al*.*^11^ investigated the dynamics of COVID-19 in Italy using Caputo fractional model. They examined that results achieved through fractional model provides a closer forecast with the real data. Higazy^12^ studied the spread of COVID-19 pandemic using SIDATHE fractional model with Caputo fractional operator. Besides this, fractional calculus is useful in various aspects of real-world problems such as Ozarslan et al*.*^13^ calculated the solutions of Malthusian growth model and falling body problem by considering the newly developed definition of Liouville–Caputo fractional conformable derivative and compared their results with the Liouville–Caputo fractional derivative. Qureshi and Aziz^14^ analyzed the solutions of chemical kinetic equation model through Laplace transform. They fractionalized their model by using Caputo operator and expressed their final solutions in the form of Mittag–Leffler function and power series expansion with double summation.

The aim of present study is to investigate the dynamics and transmission of COVID-19 through SEIR model. Furthermore, we transformed the classical model by newly developed Atangana–Baleanu time fractional model. The purpose of using AB time fractional derivative is that it has non-singular and non-local kernel and it may predict the spread of COVID-19 accurately. Qureshi et al*.*^15^ investigated the dynamics of diarrhea by considering AB fractional operator. They found that the diarrhea model under investigation estimates the real statistical data well enough when considered with the AB fractional order operator which has non-local and non-singular kernel. Bas and Ozarslan^16^ investigated the analytical solutions of some dynamical models such as logistic equation, population growth/decay equation, blood alcohol model by considering AB fractional derivative. They concluded that AB fractional operator gives more accurate results to the derivative with exponential kernel. Bas et al*.*^17^ analyzed the solutions of heating and cooling of building models by using the Laplace transform technique. They generalized their models by considering Caputo, Caputo–Fabrizio and Atangana–Baleanu fractional operators and by comparing their results, they noticed that AB-fractional derivative provides more precise results for the given models. They concluded that CF and AB fractional operators can better describes the dynamics of complex systems as compared to classical operators.

Recently, many researchers have chosen AB fractional model for dynamics of different infectious diseases, like the dynamics of HIV^18^, chicken pox^19^, hepatitis E^20^, dengue fever^21^, rubella disease^22^, measles^23^, tuberculosis^24^. Besides this, AB fractional operator is also used by many researchers to investigate the dynamics of COVID-19. Gao et al*.*^25^ reported the investigation of dynamics of COVID-19 in Wuhan, China using AB fractional model. Atangana and Araz^26^ reported a comprehensive study of spread of COVID-19 in South Africa and Turkey using Atangana-Seda numerical scheme. They generalized their classical model with AB fractional model and compared the upcoming predictions of COVID-19 in Turkey and South Africa. Mohammad and Trounev^27^ reported the numerical solutions of dynamical system of COVID-19 involving AB fractional operator. The applications of AB fractional derivative are not only limited to study the dynamics of infectious diseases but it has many applications in modern sciences and technologies e.g. nanofluids^28^, biofluids^29^, solar collectors^30^, chaotic processes^31^, electric circuits^32^ etc.

More precisely, in the present research, we have taken the data of COVID-19 from 19th March 2020 to 31st March 2020 and parameterized the given model to the real data. Positivity and boundedness of the present model is also discussed. Fixed points and basic reproduction number is estimated. Stability analysis and some basic properties have been proved for the given model. A numerical scheme is developed for the given fractional model and displayed its results for the fractional parameter $\alpha$ through graphs. Some figures are plotted with different initial conditions for global asymptotical stability (GAS).

## Mathematical modeling

**Figure 1:**
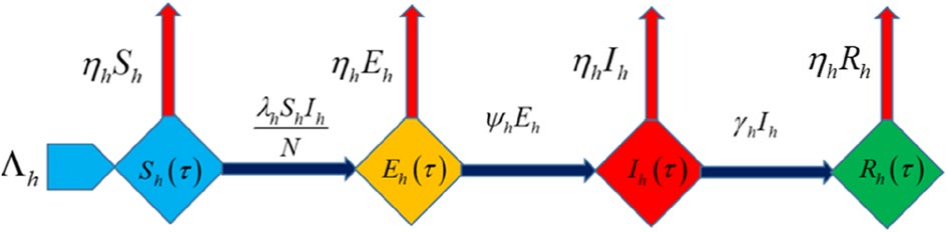
Flow chart of the given SEIR model.

In the present mathematical model, we have considered the spread of COVID-19 in human. The total population is denoted by $N_{h} (\tau )$ which is further subdivided into four sub-classes. The four subclasses are susceptible, exposed, infected and recovered people which are denoted by $S_{h} (\tau ),\,\,E_{h} (\tau ),\,\,I_{h} (\tau )\,\,{\text{and}}\,\,R_{h} (\tau )$ respectively. The interaction among these subclasses has shown in the flow chart which is Fig. 1.

In the given model, the recruitment rate of susceptible population is $\Lambda_{h}$ which represents birth rate,$\eta_{h}$ represents death rate of each subclass, $\lambda_{h}$ represents interaction rate between susceptible and infected population with the route $\frac{{\lambda_{h} S_{h} I_{h} }}{N}$, $\psi_{h}$ represents the rate at which exposed class completed their incubation period and enter into the infected class, $\gamma_{h}$ is the removal or recovery rate of infected population.

1 $$\left. \begin{gathered} \frac{{dS_{h} (\tau )}}{d\tau } = \Lambda_{h} - \eta_{h} S_{h} - \frac{{\lambda_{h} S_{h} I_{h} }}{N}, \hfill \\ \frac{{dE_{h} (\tau )}}{d\tau } = \frac{{\lambda_{h} S_{h} I_{h} }}{N} - \eta_{h} E_{h} - \psi_{h} E_{h} , \hfill \\ \frac{{dI_{h} (\tau )}}{d\tau } = \psi_{h} E_{h} - \eta_{h} I_{h} - \gamma_{h} I_{h} , \hfill \\ \frac{{dR_{h} (\tau )}}{d\tau } = \gamma_{h} I_{h} - \eta_{h} R_{h} , \hfill \\ \end{gathered} \right\}$$

Corresponding non-negative initial conditions

2 $$S(0) = S^{*} \ge 0,\;\;E(0) = E^{*} \ge 0,\;\;I(0) = I^{*} \ge 0\quad {\text{and}}\quad R(0) = R^{*} \ge 0.$$

## Non-negativity and boundedness of the model

This section provided to prove the boundedness and positivity of the solutions of system (1). To show the positivity of the model (1), we state the following lemma:

### Lemma 3.1

*Suppose *$\Phi \subset {\mathbb{R}} \times {\mathbb{C}}^{n}$* is open, *$g_{i} \in {\mathbb{C}}(\Phi ,\;{\mathbb{R}}),\;\;i = 1,\;2,\;3, \ldots ,\;n.$* If *$\left. {g_{i} } \right|_{{x_{i} (\tau ) = 0,X_{\tau } \in \;{\mathbb{C}}_{ + 0}^{n} }} \ge 0$, $X_{\tau } = (x_{1\tau } ,\;x_{2\tau } , \ldots ,\;x_{n\tau } )^{T} ,\;i = 1,\;2, \ldots ,\;n$,* then *${\mathbb{C}}_{ + 0}^{n} \left\{ {\varphi = (\varphi_{1} ,\;\varphi_{2} , \ldots ,\;\varphi_{n} ):\varphi \in {\mathbb{C}}([ - \upsilon ,\;0],\;{\mathbb{R}}_{ + 0}^{n} )} \right\}$* is the invariant domain of the following equations.*

3 $$\frac{{dx_{i} (\tau )}}{d\tau } = g_{i} (\tau ,\;X_{\tau } ),\quad \tau \ge \sigma ,\;\;i = 1,\;2, \ldots ,\;n.$$

where

4 $${\mathbb{R}}_{ + 0}^{n} \left\{ {(x_{1} ,\;x_{2} , \ldots ,\;x_{n} ):x_{i} \ge 0,\quad i = 1,\;2, \ldots ,\;n} \right\}$$

### Preposition 3.1

*The system* (1) *is invariant in *${\mathbb{R}}_{ + }^{4}$*.*

### *Proof*

By rewriting the system (1), we have:

5 $$\frac{dX}{{d\tau }} = M(X(\tau )),\quad X(0) = X_{0} \ge 0,$$

6 $$M(X(\tau )) = \left( {M_{1} (X),\;M_{2} (X),\;M_{3} (X),\;M_{4} (X)} \right)^{T} .$$

We noted that

7 $$\left. \begin{gathered} \left. {\frac{{dS_{h} (\tau )}}{d\tau }} \right|_{{S_{h} = 0}} = \Lambda_{h} \ge 0 \hfill \\ \left. {\frac{{dE_{h} (\tau )}}{d\tau }} \right|_{{E_{h} = 0}} = \frac{{\lambda_{h} S_{h} I_{h} }}{{S_{h} + I_{h} + R_{h} }} \ge 0 \hfill \\ \left. {\frac{{dI_{h} (\tau )}}{d\tau }} \right|_{{I_{h} = 0}} = \psi_{h} E_{h} \ge 0 \hfill \\ \left. {\frac{{dR_{h} (\tau )}}{d\tau }} \right|_{{R_{h} = 0}} = \gamma_{h} I_{h} \ge 0 \hfill \\ \end{gathered} \right\},$$

According to Lemma 3.1, ${\mathbb{R}}_{ + }^{4}$ is invariant set.

### Preposition 3.2

*The system* (1) *is bounded in the region:*

$$\Phi = \left\{ {\left( {S_{h} (\tau ),\;E_{h} (\tau ),\;I_{h} (\tau ),\;R_{h} (\tau )} \right) \in {\mathbb{R}}^{4} :N_{h} (\tau ) \le \frac{{\Lambda_{h} }}{{\eta_{h} }}} \right\}.$$

### *Proof*

Boundedness of the problem (1) can be verified by adding all equations of system (1), we obtained:

8 $$\frac{{dN_{h} (\tau )}}{d\tau } = \Lambda_{h} - \eta_{h} N_{h} ,\quad {\text{with}}\quad N_{h} (0) = N_{0} \ge 0.$$

The solution of Eq. (8) takes the form:

9 $$N_{h} (\tau ) \le N_{0} e^{{ - \eta_{h} \tau }} + \frac{{\Lambda_{h} }}{{\eta_{h} }}(1 - e^{{ - \eta_{h} \tau }} ).$$

It can be clearly seen from Eq. (9) that if $\tau \to \infty$ then $N_{h} (\tau ) \le \frac{{\Lambda_{h} }}{{\eta_{h} }}$ which means that the feasible region for the given model will be:

10 $$\Phi = \left\{ {\left( {S_{h} (\tau ),\;E_{h} (\tau ),\;I_{h} (\tau ),\;R_{h} (\tau )} \right) \in {\mathbb{R}}^{4} :N_{h} (\tau ) \le \frac{{\Lambda_{h} }}{{\eta_{h} }}} \right\}.$$

Hence the solution of the system (1) is bounded.

## Equilibrium points, basic reproduction number and local stability analysis

This section includes the possible fixed points of model (1). There exists two possible equilibrium points are calculated, i.e. Disease-free equilibrium (DFE) and endemic equilibrium (EE). Furthermore, basic reproduction number is calculated by next generation technique and discuss the local stable analysis of these equilibrium points. The steady state solution of model is given below by considering the rate of change w.r.t time becomes zero:

11 $$\left. {\frac{{dS_{h} (\tau )}}{d\tau } = \frac{{dE_{h} (\tau )}}{d\tau } = \frac{{dI_{h} (\tau )}}{d\tau } = \frac{{dR_{h} (\tau )}}{d\tau } = 0} \right\},$$

Using Eq. (11), model (1) becomes:

12 $$\left. \begin{gathered} 0 = \Lambda_{h} - \eta_{h} S_{h} - \frac{{\lambda_{h} S_{h} I_{h} }}{N}, \hfill \\ 0 = \frac{{\lambda_{h} S_{h} I_{h} }}{N} - \eta_{h} E_{h} - \psi_{h} E_{h} , \hfill \\ 0 = \psi_{h} E_{h} - \eta_{h} I_{h} - \gamma_{h} I_{h} , \hfill \\ 0 = \gamma_{h} I_{h} - \eta_{h} R_{h} , \hfill \\ \end{gathered} \right\},$$

From steady state system (12), DFE can be obtained by assuming $E_{h} = I_{h} = R_{h} = 0$ and is denoted by

13 $$\Psi_{DFE} = (S^{0} ,\;E^{0} ,\;I^{0} ,\;R^{0} ) = \left( {\frac{{\Lambda_{h} }}{{\eta_{h} }},\;0,\;0,\;0} \right).$$

Similarly, EE of model (1) is obtained from system (12) and is given by: $\Psi_{EE} = (S^{*} ,\;E^{*} ,\;I^{*} ,\;R^{*} )$, where,

14 $$\left. \begin{gathered} S^{*} = \frac{{\Lambda_{h} \left( {\eta_{h}^{2} - \gamma_{h} \psi_{h} - \eta_{h} \psi_{h} } \right)}}{{\eta_{h} \left( {\eta_{h}^{2} + \eta_{h} \gamma_{h} + \eta_{h} \lambda_{h} - \eta_{h} \psi_{h} - \lambda_{h} \psi_{h} } \right)}}, \hfill \\ E^{*} = \frac{{\Lambda_{h} \left( {\eta_{h} \gamma_{h} + \eta_{h} \lambda_{h} + \gamma_{h} \psi_{h} - \lambda_{h} \psi_{h} } \right)}}{{(\eta_{h} + \psi_{h} )\left( {\eta_{h}^{2} + \eta_{h} \gamma_{h} + \eta_{h} \lambda_{h} - \eta_{h} \psi_{h} - \lambda_{h} \psi_{h} } \right)}}, \hfill \\ I^{*} = \frac{{\Lambda_{h} (\psi_{h} - \eta_{h} )\left( {\eta_{h} \gamma_{h} + \eta_{h} \lambda_{h} + \gamma_{h} \psi_{h} - \lambda_{h} \psi_{h} } \right)}}{{\gamma_{h} (\eta_{h} + \psi_{h} )\left( {\eta_{h}^{2} + \eta_{h} \gamma_{h} + \eta_{h} \lambda_{h} - \eta_{h} \psi_{h} - \lambda_{h} \psi_{h} } \right)}}, \hfill \\ R^{*} = \frac{{\Lambda_{h} (\psi_{h} - \eta_{h} )\left( {\eta_{h} \gamma_{h} + \eta_{h} \lambda_{h} + \gamma_{h} \psi_{h} - \lambda_{h} \psi_{h} } \right)}}{{\eta_{h} (\eta_{h} + \psi_{h} )\left( {\eta_{h}^{2} + \eta_{h} \gamma_{h} + \eta_{h} \lambda_{h} - \eta_{h} \psi_{h} - \lambda_{h} \psi_{h} } \right)}}, \hfill \\ \end{gathered} \right\}$$

The basic reproduction number ${\mathfrak{R}}_{0}$ is calculated by next generation technique^33^. The *F* and *V* matrices at DFE $\Psi^{0}$ is given as follows:

15 $$F = \left[ {\begin{array}{*{20}c} 0 & {\quad \lambda_{h} } \\ 0 & {\quad 0} \\ \end{array} } \right],$$

16 $$V = \left[ {\begin{array}{*{20}c} {\eta_{h} + \psi_{h} } & {\quad 0_{h} } \\ {\psi_{h} } & {\quad \eta_{h} + \gamma_{h} } \\ \end{array} } \right],$$

According to^33^ the spectral radius $\rho (FV^{ - 1} )$ is the required reproduction number ${\mathfrak{R}}_{0}$ which is calculated in the form:

17 $${\mathfrak{R}}_{0} = \frac{{\lambda_{h} \psi_{h} }}{{(\eta_{h} + \psi_{h} )(\eta_{h} + \gamma_{h} )}}.$$

### Theorem 4.1

*The DFE *$\Psi^{0}$* of the system* (1) *is locally asymptotically stable if *${\mathfrak{R}}_{0} < 1$*.*

### *Proof*

The Jacobian matrix of system (1) at DFE is given by:

18 $$J_{{\Psi^{0} }} = \left[ {\begin{array}{*{20}c} { - \eta_{h} } & {\quad 0} & {\quad - \lambda_{h} } & {\quad 0} \\ 0 & {\quad - \eta_{h} - \psi_{h} } & {\quad \lambda } & {\quad 0} \\ 0 & {\quad \psi_{h} } & {\quad - \eta_{h} - \gamma_{h} } & {\quad 0} \\ 0 & {\quad 0} & {\quad \gamma_{h} } & {\quad - \eta_{h} } \\ \end{array} } \right],$$

Suppose $\lambda$ denote the eigen values of the Jacobian matrix $J_{{\Psi^{0} }}$. Here, the two eigen values of the above matrix are negative i.e. $- \;\eta_{h}$ (twice). The remaining required eigen values can be obtained by the following characteristic equation:

19 $$\lambda^{2} + \kappa_{1} \lambda + \kappa_{2} = 0,$$

where

20 $$\kappa_{1} = \gamma_{h} + 2\eta_{h} + \psi_{h} ,$$

21 $$\begin{aligned} \kappa_{2} & = \eta_{h} \gamma + \eta_{h}^{2} + \gamma_{h} \psi_{h} - \lambda_{h} \psi_{h} , \\ & \Rightarrow \kappa_{2} = \left( {\eta_{h} + \gamma_{h} } \right)\left( {\eta_{h} + \psi_{h} } \right)\left\{ {1 - {\mathfrak{R}}_{0} } \right\}. \\ \end{aligned}$$

From Eq. (17), it can be clearly noticed that $\kappa_{1} > 0$. Similarly, from Eq. (18), it can be noticed that $\kappa_{2} > 0$ when ${\mathfrak{R}}_{0} < 1$. So, all the coefficients of characteristics equation are non-negative. Furthermore, according to Rough-Hurtwiz criteria, the eigen values of the above characteristics equation are negative. Thus, all the eigen values of Jacobian matrix (15) are negative for ${\mathfrak{R}}_{0} < 1$. Hence the model (1) is locally asymptotically stable when ${\mathfrak{R}}_{0} < 1$.

## Fractional model and numerical scheme

### Preliminaries

#### Definition 5.1

The Atangana–Baleanu (AB) time fractional derivative with fractional order $\alpha$ is defined as^34^.

22 $${}_{a}^{AB} \wp_{\tau }^{\alpha } f(\tau) = \frac{{\mathfrak{I}} (\alpha )}{{1 - \alpha }}\int\limits_{a}^{\tau } {E_{a} \left( {\frac{{ - \alpha (\tau - t)^{\alpha } }}{1 - \alpha }} \right)} f^{^{\prime}} (\tau )dt,\quad {\text{for}}\;\;0 < \alpha < 1.$$

Here, ${\mathfrak{I}} (\alpha )$ is the normalization function and $E_{\alpha } (.)$ is Mittage–Leffler function^35^.

#### Definition 5.2

The numerical scheme for the solution of fractional order ODE is defined by Toufik and Atangana^36^:

Consider a non-linear fractional ODE:

23 $${}^{AB}\wp_{t }^{\alpha } y(t) = f(t,\;y(t))\quad {\text{with}}\;\; \, y(0) = y_{0} ,$$

The numerical scheme for Eq. (23) is defined as^36^:

24 $$\begin{aligned} y_{n + 1} & = y_{0} + \frac{1 - \alpha }{{{\mathfrak{I}} (\alpha )}}f(t_{n} ,\;y(t_{n} )) \\ & \quad + \frac{\alpha }{{\mathfrak{I}} (\alpha )}\sum\limits_{k = 0}^{n} {\left[ \begin{gathered} \frac{{h^{\alpha } f(t_{k} ,\;y(t_{k} ))}}{\Gamma (\alpha + 2)}\left\{ {(n + 1 - k)^{\alpha } (n + 2 - k + \alpha ) - (n - k)^{\alpha } (n + 2 - k + 2\alpha )} \right\} \hfill \\ - \frac{{h^{\alpha } f(t_{k - 1} ,\;y(t_{k - 1} ))}}{\Gamma (\alpha + 2)}\left\{ {(n + 1 - k)^{\alpha + 1} - (n - k)^{\alpha } (n + 1 - k + \alpha )} \right\} \hfill \\ \end{gathered} \right]} . \\ \end{aligned}$$

### Fractional model

For the generalization of model (1), we replace classical time derivative with Atangana–Baleanu time fractional derivative, model (1) takes the following generalized form:

25 $$\left. \begin{gathered} {}_{0}^{AB} \wp_{\tau }^{\alpha } S_{h} (\tau ) = \Lambda_{h} - \eta_{h} S_{h} - \frac{{\lambda_{h} S_{h} I_{h} }}{N}, \hfill \\ {}_{0}^{AB} \wp_{\tau }^{\alpha } E_{h} (\tau ) = \frac{{\lambda_{h} S_{h} I_{h} }}{N} - \eta_{h} E_{h} - \psi_{h} E_{h} , \hfill \\ {}_{0}^{AB} \wp_{\tau }^{\alpha } I_{h} (\tau ) = \psi_{h} E_{h} - \eta_{h} I_{h} - \gamma_{h} I_{h} , \hfill \\ {}_{0}^{AB} \wp_{\tau }^{\alpha } R_{h} (\tau ) = \gamma_{h} I_{h} - \eta_{h} R_{h} , \hfill \\ \end{gathered} \right\},$$

here, $\alpha$ is fractional parameter and ${}_{0}^{AB} \wp_{\tau }^{\alpha } (.)$ is the AB time fractional derivative. All these variables and initial conditions for model (25) are positive.

### Numerical scheme

Adopting the procedure in^36^, model (25) takes the following appropriate form:

26 $$\left. \begin{gathered} {}_{0}^{AB} \wp_{\tau }^{\alpha } S_{h} (\tau ) = \chi_{1} \left( {t,\;S_{h} ,\;E_{h} ,\;I_{h} ,\;R_{h} } \right), \hfill \\ {}_{0}^{AB} \wp_{\tau }^{\alpha } E_{h} (\tau ) = \chi_{2} \left( {t,\;S_{h} ,\;E_{h} ,\;I_{h} ,\;R_{h} } \right), \hfill \\ {}_{0}^{AB} \wp_{\tau }^{\alpha } I_{h} (\tau ) = \chi_{3} \left( {t,\;S_{h} ,\;E_{h} ,\;I_{h} ,\;R_{h} } \right), \hfill \\ {}_{0}^{AB} \wp_{\tau }^{\alpha } R_{h} (\tau ) = \chi_{4} \left( {t,\;S_{h} ,\;E_{h} ,\;I_{h} ,\;R_{h} } \right), \hfill \\ \end{gathered} \right\}.$$

Furthermore, system (26) becomes:

27 $$S_{h} (\tau ) - S_{h} (0) = \frac{1 - \alpha }{{{\mathfrak{I}} (\alpha )}}\chi_{1} (\tau ,\;S_{h} ,\;E_{h} ,\;I_{h} ,\;R_{h} ) + \frac{\alpha }{{\mathfrak{I}} (\alpha )\Gamma (\alpha )}\int\limits_{0}^{\tau } {\chi_{1} (\zeta ,\;S_{h} ,\;E_{h} ,\;I_{h} ,\;R_{h} )} (\tau - \zeta )^{\alpha - 1} d\zeta ,$$

28 $$E_{h} (\tau ) - E_{h} (0) = \frac{1 - \alpha }{{{\mathfrak{I}} (\alpha )}}\chi_{2} (\tau ,\;S_{h} ,\;E_{h} ,\;I_{h} ,\;R_{h} ) + \frac{\alpha }{{\mathfrak{I}} (\alpha )\Gamma (\alpha )}\int\limits_{0}^{\tau } {\chi_{2} (\zeta ,\;S_{h} ,\;E_{h} ,\;I_{h} ,\;R_{h} )} (\tau - \zeta )^{\alpha - 1} d\zeta ,$$

29 $$I_{h} (\tau ) - I_{h} (0) = \frac{1 - \alpha }{{{\mathfrak{I}} (\alpha )}}\chi_{3} (\tau ,\;S_{h} ,\;E_{h} ,\;I_{h} ,\;R_{h} ) + \frac{\alpha }{{\mathfrak{I}} (\alpha )\Gamma (\alpha )}\int\limits_{0}^{\tau } {\chi_{3} (\zeta ,\;S_{h} ,\;E_{h} ,\;I_{h} ,\;R_{h} )} (\tau - \zeta )^{\alpha - 1} d\zeta ,$$

30 $$R_{h} (\tau ) - R_{h} (0) = \frac{1 - \alpha }{{{\mathfrak{I}} (\alpha )}}\chi_{4} (\tau ,\;S_{h} ,\;E_{h} ,\;I_{h} ,\;R_{h} ) + \frac{\alpha }{{\mathfrak{I}} (\alpha )\Gamma (\alpha )}\int\limits_{0}^{\tau } {\chi_{4} (\zeta ,\;S_{h} ,\;E_{h} ,\;I_{h} ,\;R_{h} )} (\tau - \zeta )^{\alpha - 1} d\zeta ,$$

Now, taking $\tau = \tau_{n + 1}$, $n = 1,\;2, \ldots$**,** Eqs. (27)–(30) adopt the following shape:

31 $$\begin{aligned} S(\tau_{n + 1} ) - S(\tau_{0} ) & = \frac{1 - \alpha }{{{\mathfrak{I}} (\alpha )}}\chi_{1} (\tau_{n} ,\;S_{h} ,\;E_{h} ,\;I_{h} ,\;R_{h} ) \\ & \quad + \frac{\alpha }{{\mathfrak{I}} (\alpha )\Gamma (\alpha )}\sum\limits_{m = 0}^{n} {\int\limits_{{\tau_{m} }}^{{\tau_{m + 1} }} {\chi_{1} (\zeta ,\;S_{h} ,\;E_{h} ,\;I_{h} ,\;R_{h} )} (\tau_{n + 1} - \zeta )^{\alpha - 1} d\zeta } , \\ \end{aligned}$$

32 $$\begin{aligned} E(\tau_{n + 1} ) - E(\tau_{0} ) & = \frac{1 - \alpha }{{{\mathfrak{I}} (\alpha )}}\chi_{2} (\tau_{n} ,\;S_{h} ,\;E_{h} ,\;I_{h} ,\;R_{h} ) \\ & \quad + \frac{\alpha }{{\mathfrak{I}} (\alpha )\Gamma (\alpha )}\sum\limits_{m = 0}^{n} {\int\limits_{{\tau_{m} }}^{{\tau_{m + 1} }} {\chi_{2} (\zeta ,\;S_{h} ,\;E_{h} ,\;I_{h} ,\;R_{h} )} (\tau_{n + 1} - \zeta )^{\alpha - 1} d\zeta } , \\ \end{aligned}$$

33 $$\begin{aligned} I(\tau_{n + 1} ) - I(\tau_{0} ) & = \frac{1 - \alpha }{{{\mathfrak{I}} (\alpha )}}\chi_{3} (\tau_{n} ,\;S_{h} ,\;E_{h} ,\;I_{h} ,\;R_{h} ) \\ & \quad + \frac{\alpha }{{\mathfrak{I}} (\alpha )\Gamma (\alpha )}\sum\limits_{m = 0}^{n} {\int\limits_{{\tau_{m} }}^{{\tau_{m + 1} }} {\chi_{3} (\zeta ,\;S_{h} ,\;E_{h} ,\;I_{h} ,\;R_{h} )} (\tau_{n + 1} - \zeta )^{\alpha - 1} d\zeta } , \\ \end{aligned}$$

34 $$\begin{aligned} R(\tau_{n + 1} ) - R(\tau_{0} ) & = \frac{1 - \alpha }{{{\mathfrak{I}} (\alpha )}}\chi_{4} (\tau_{n} ,\;S_{h} ,\;E_{h} ,\;I_{h} ,\;R_{h} ) \\ & \quad + \frac{\alpha }{{\mathfrak{I}} (\alpha )\Gamma (\alpha )}\sum\limits_{m = 0}^{n} {\int\limits_{{\tau_{m} }}^{{\tau_{m + 1} }} {\chi_{4} (\zeta ,\;S_{h} ,\;E_{h} ,\;I_{h} ,\;R_{h} )} (\tau_{n + 1} - \zeta )^{\alpha - 1} d\zeta } , \\ \end{aligned}$$

Assuming the interval $[\tau_{m} ,\;\tau_{m + 1} ]$ and using the two-step Lagrange polynomial interpolation, Eqs. (31)–(34) becomes:

35 $$\begin{aligned} S(\tau_{n + 1} ) - S(\tau_{0} ) & = \frac{1 - \alpha }{{{\mathfrak{I}} (\alpha )}}\chi_{1} (\tau_{n} ,\;S_{h} ,\;E_{h} ,\;I_{h} ,\;R_{h} ) \\ & \quad + \frac{\alpha }{{\mathfrak{I}} (\alpha )\Gamma (\alpha )}\sum\limits_{m = 0}^{n} {\left( \begin{gathered} \frac{{\chi_{1} (\tau_{m} ,\;S_{h} ,\;E_{h} ,\;I_{h} ,\;R_{h} )}}{l}\int\limits_{{\tau_{m} }}^{{\tau_{m + 1} }} {(\zeta - \tau_{m + 1} )^{\alpha - 1} (\tau_{n + 1} - \zeta )^{\alpha - 1} } d\zeta \hfill \\ - \frac{{\chi_{1} (\tau_{m - 1} ,\;S_{h} ,\;E_{h} ,\;I_{h} ,\;R_{h} )}}{l}\int\limits_{{\tau_{m} }}^{{\tau_{m + 1} }} {(\zeta - \tau_{m} )^{\alpha - 1} (\tau_{n + 1} - \zeta )^{\alpha - 1} } d\zeta \hfill \\ \end{gathered} \right),} \\ \end{aligned}$$

36 $$\begin{aligned} E(\tau_{n + 1} ) - E(\tau_{0} ) & = \frac{1 - \alpha }{{{\mathfrak{I}} (\alpha )}}\chi_{2} (\tau_{n} ,\;S_{h} ,\;E_{h} ,\;I_{h} ,\;R_{h} ) \\ & \quad + \frac{\alpha }{{\mathfrak{I}} (\alpha )\Gamma (\alpha )}\sum\limits_{m = 0}^{n} {\left( \begin{gathered} \frac{{\chi_{2} (\tau_{m} ,\;S_{h} ,\;E_{h} ,\;I_{h} ,\;R_{h} )}}{l}\int\limits_{{\tau_{m} }}^{{\tau_{m + 1} }} {(\zeta - \tau_{m + 1} )^{\alpha - 1} (\tau_{n + 1} - \zeta )^{\alpha - 1} } d\zeta \hfill \\ - \frac{{\chi_{2} (\tau_{m - 1} ,\;S_{h} ,\;E_{h} ,\;I_{h} ,\;R_{h} )}}{l}\int\limits_{{\tau_{m} }}^{{\tau_{m + 1} }} {(\zeta - \tau_{m} )^{\alpha - 1} (\tau_{n + 1} - \zeta )^{\alpha - 1} } d\zeta \hfill \\ \end{gathered} \right)} , \\ \end{aligned}$$

37 $$\begin{aligned} I(\tau_{n + 1} ) - I(\tau_{0} ) & = \frac{1 - \alpha }{{{\mathfrak{I}} (\alpha )}}\chi_{1} (\tau_{n} ,\;S_{h} ,\;E_{h} ,\;I_{h} ,\;R_{h} ) \\ & \quad + \frac{\alpha }{{\mathfrak{I}} (\alpha )\Gamma (\alpha )}\sum\limits_{m = 0}^{n} {\left( \begin{gathered} \frac{{\chi_{3} (\tau_{m} ,\;S_{h} ,\;E_{h} ,\;I_{h} ,\;R_{h} )}}{l}\int\limits_{{\tau_{m} }}^{{\tau_{m + 1} }} {(\zeta - \tau_{m + 1} )^{\alpha - 1} (\tau_{n + 1} - \zeta )^{\alpha - 1} } d\zeta \hfill \\ - \frac{{\chi_{3} (\tau_{m - 1} ,\;S_{h} ,\;E_{h} ,\;I_{h} ,\;R_{h} )}}{l}\int\limits_{{\tau_{m} }}^{{\tau_{m + 1} }} {(\zeta - \tau_{m} )^{\alpha - 1} (\tau_{n + 1} - \zeta )^{\alpha - 1} } d\zeta \hfill \\ \end{gathered} \right)} , \\ \end{aligned}$$

38 $$\begin{aligned} R(\tau_{n + 1} ) - R(\tau_{0} ) & = \frac{1 - \alpha }{{{\mathfrak{I}} (\alpha )}}\chi_{1} (\tau_{n} ,\;S_{h} ,\;E_{h} ,\;I_{h} ,\;R_{h} ) \\ & \quad + \frac{\alpha }{{\mathfrak{I}} (\alpha )\Gamma (\alpha )}\sum\limits_{m = 0}^{n} {\left( \begin{gathered} \frac{{\chi_{4} (\tau_{m} ,\;S_{h} ,\;E_{h} ,\;I_{h} ,\;R_{h} )}}{l}\int\limits_{{\tau_{m} }}^{{\tau_{m + 1} }} {(\zeta - \tau_{m + 1} )^{\alpha - 1} (\tau_{n + 1} - \zeta )^{\alpha - 1} } d\zeta \hfill \\ - \frac{{\chi_{4} (\tau_{m - 1} ,\;S_{h} ,\;E_{h} ,\;I_{h} ,\;R_{h} )}}{l}\int\limits_{{\tau_{m} }}^{{\tau_{m + 1} }} {(\zeta - \tau_{m} )^{\alpha - 1} (\tau_{n + 1} - \zeta )^{\alpha - 1} } d\zeta \hfill \\ \end{gathered} \right)} . \\ \end{aligned}$$

where $l$ is the step size.

By solving the integration terms in the above equations, Eqs. (35)–(38) gets the following iterative shape:

39 $$\begin{aligned} S(\tau_{n + 1} ) & = S(\tau_{0} ) + \frac{1 - \alpha }{{{\mathfrak{I}} (\alpha )}}\chi_{1} (t_{n} ,\;S_{h} ,\;E_{h} ,\;I_{h} ,\;R_{h} ) \\ & \quad + \frac{\alpha }{{\mathfrak{I}} (\alpha )}\sum\limits_{m = 0}^{n} {\left[ \begin{gathered} \frac{{l^{\alpha } \chi_{1} (t_{m} ,\;S_{h} ,\;E_{h} ,\;I_{h} ,\;R_{h} )}}{\Gamma (\alpha + 2)}\left\{ {(n + 1 - m)^{\alpha } (n + 2 - m + \alpha ) - (n - m)^{\alpha } (n + 2 - m + 2\alpha )} \right\} \hfill \\ - \frac{{l^{\alpha } \chi_{1} (t_{m - 1} ,\;S_{h} ,\;E_{h} ,\;I_{h} ,\;R_{h} )}}{\Gamma (\alpha + 2)}\left\{ {(n + 1 - m)^{\alpha + 1} - (n - m)^{\alpha } (n + 1 - m + \alpha )} \right\} \hfill \\ \end{gathered} \right]} , \\ \end{aligned}$$

40 $$\begin{aligned} E(\tau_{n + 1} ) & = E(\tau_{0} ) + \frac{1 - \alpha }{{{\mathfrak{I}} (\alpha )}}\chi_{2} (t_{n} ,\;S_{h} ,\;E_{h} ,\;I_{h} ,\;R_{h} ) \\ & \quad + \frac{\alpha }{{\mathfrak{I}} (\alpha )}\sum\limits_{m = 0}^{n} {\left[ \begin{gathered} \frac{{l^{\alpha } \chi_{2} (t_{m} ,\;S_{h} ,\;E_{h} ,\;I_{h} ,\;R_{h} )}}{\Gamma (\alpha + 2)}\left\{ {(n + 1 - m)^{\alpha } (n + 2 - m + \alpha ) - (n - m)^{\alpha } (n + 2 - m + 2\alpha )} \right\} \hfill \\ - \frac{{l^{\alpha } \chi_{2} \left( {t_{m - 1} ,\;S_{h} ,\;E_{h} ,\;I_{h} ,\;R_{h} } \right)}}{\Gamma (\alpha + 2)}\left\{ {(n + 1 - m)^{\alpha + 1} - (n - m)^{\alpha } (n + 1 - m + \alpha )} \right\} \hfill \\ \end{gathered} \right]} , \\ \end{aligned}$$

41 $$\begin{aligned} I(\tau_{n + 1} ) & = I(\tau_{0} ) + \frac{1 - \alpha }{{{\mathfrak{I}} (\alpha )}}\chi_{3} (t_{n} ,\;S_{h} ,\;E_{h} ,\;I_{h} ,\;R_{h} ) \\ & \quad + \frac{\alpha }{{\mathfrak{I}} (\alpha )}\sum\limits_{m = 0}^{n} {\left[ \begin{gathered} \frac{{l^{\alpha } \chi_{3} (t_{m} ,\;S_{h} ,\;E_{h} ,\;I_{h} ,\;R_{h} )}}{\Gamma (\alpha + 2)}\left\{ {(n + 1 - m)^{\alpha } (n + 2 - m + \alpha ) - (n - m)^{\alpha } (n + 2 - m + 2\alpha )} \right\} \hfill \\ - \frac{{l^{\alpha } \chi_{3} (t_{m - 1} ,\;S_{h} ,\;E_{h} ,\;I_{h} ,\;R_{h} )}}{\Gamma (\alpha + 2)}\left\{ {(n + 1 - m)^{\alpha + 1} - (n - m)^{\alpha } (n + 1 - m + \alpha )} \right\} \hfill \\ \end{gathered} \right]} , \\ \end{aligned}$$

42 $$\begin{aligned} R(\tau_{n + 1} ) & = R(\tau_{0} ) + \frac{1 - \alpha }{{{\mathfrak{I}} (\alpha )}}\chi_{4} (t_{n} ,\;S_{h} ,\;E_{h} ,\;I_{h} ,\;R_{h} ) \\ & \quad + \frac{\alpha }{{\mathfrak{I}} (\alpha )}\sum\limits_{m = 0}^{n} {\left[ \begin{gathered} \frac{{l^{\alpha } \chi_{4} (t_{m} ,\;S_{h} ,\;E_{h} ,\;I_{h} ,\;R_{h} )}}{\Gamma (\alpha + 2)}\left\{ {(n + 1 - m)^{\alpha } (n + 2 - m + \alpha ) - (n - m)^{\alpha } (n + 2 - m + 2\alpha )} \right\} \hfill \\ - \frac{{l^{\alpha } \chi_{4} (t_{m - 1} ,\;S_{h} ,\;E_{h} ,\;I_{h} ,\;R_{h} )}}{\Gamma (\alpha + 2)}\left\{ {(n + 1 - m)^{\alpha + 1} - (n - m)^{\alpha } (n + 1 - m + \alpha )} \right\} \hfill \\ \end{gathered} \right]} . \\ \end{aligned}$$

## Data fitting and numerical results

### Data fitting

For data fitting of model (25) we have taken some values of parameters from the literature and the remaining values are fitted for the data collected for Pakistan. We have fitted our model solutions with the real data collected from WHO for Pakistan from 19th March 2020 to 31st March 2020^5^. According to the publically reported data, the total population of Pakistan for the year 2020 is 220,410,553^37^. For the initial values we have considered $N(0) = 220{,}410{,}553$ in which the infected individuals are $I_{h} (0) = 2039$ and the exposed individuals are $E_{h} (0) = 10{,}000$. We have combined the recovered and removed population in only one class and denoted by $R_{h} (\tau )$ so that $R_{h} (0) = {\text{dead}} + {\text{recovered}} = 27 + 99 = 126$. The remaining population is considered as susceptible individuals so that $S_{h} (0) = 220{,}398{,}388$. In Pakistan, for the year 2020 the life expectancy is $67.4$ so that $\eta_{h} = \frac{1}{67.4}$^38^. The corresponding birth rate in Pakistan is estimated as $\Lambda_{h} = N(0) \times \eta_{h}$ which becomes $\Lambda_{h} = 3{,}270{,}186.2462$. Incorporating these values and after model simulation, we get different parameter values as shown in Table 1. The given model is fitted for $\alpha = 1$. We have estimated the basic reproduction number ${\mathfrak{R}}_{0} \approx 2.30748$ using different parameter values given Table 1.

**Table 1:** Estimated and fitted values for different parameters of model 1.

Parameter	Description	Value	Source
$\Lambda_{h}$	Birth rate	3,270,186.2462	Estimated
$\eta_{h}$	Natural mortality rate	$1/67.4$	^33^
$\lambda_{{_{h} }}$	Contact rate	0.29	Fitted
$\psi_{h}$	Incubation period	0.1243	Fitted
$\gamma_{h}$	Recovery rate	0.09722	Fitted

### Numerical results

**Figure 2:**
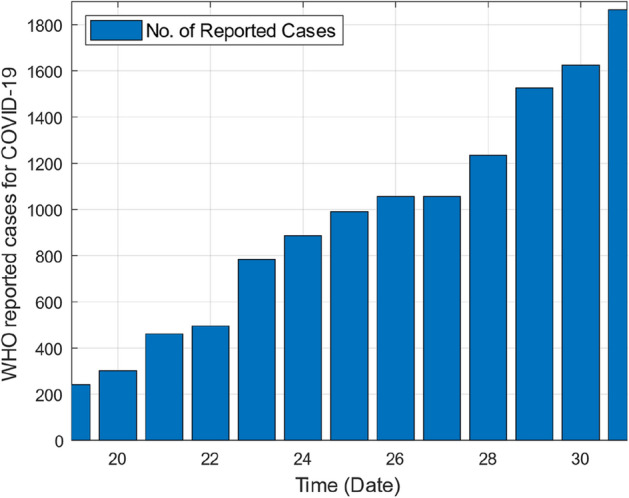
WHO reported cases for COVID-19 from 19th March 2020 till 31st March 2020.

**Figure 3:**
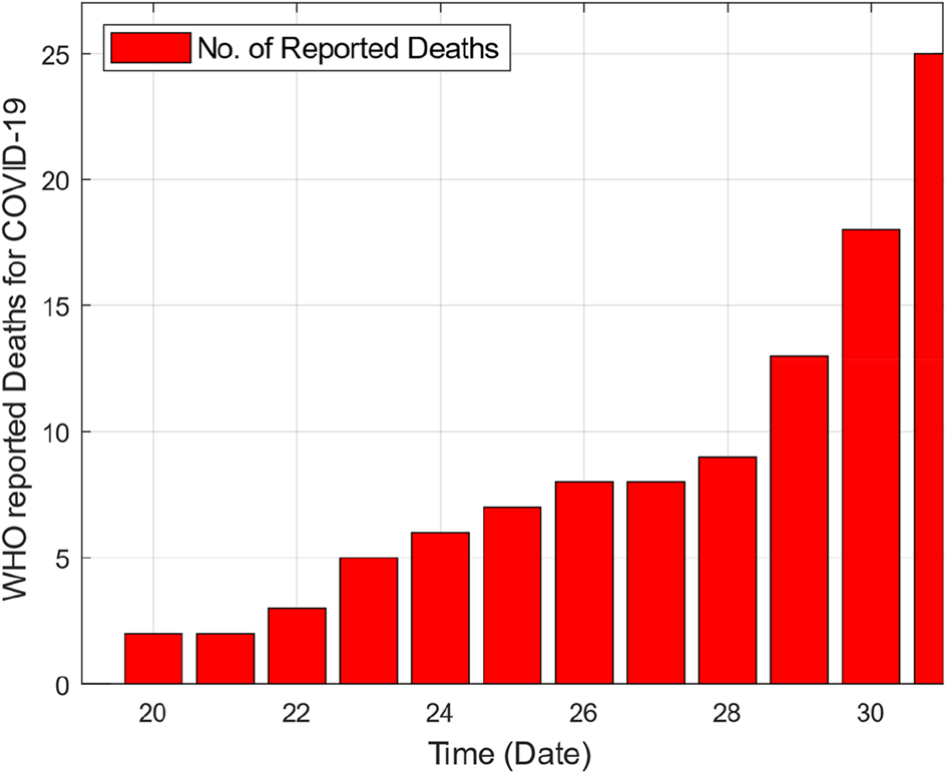
WHO reported deaths by COVID-19 from 19th March 2020 till 31st March 2020.

**Figure 4:**
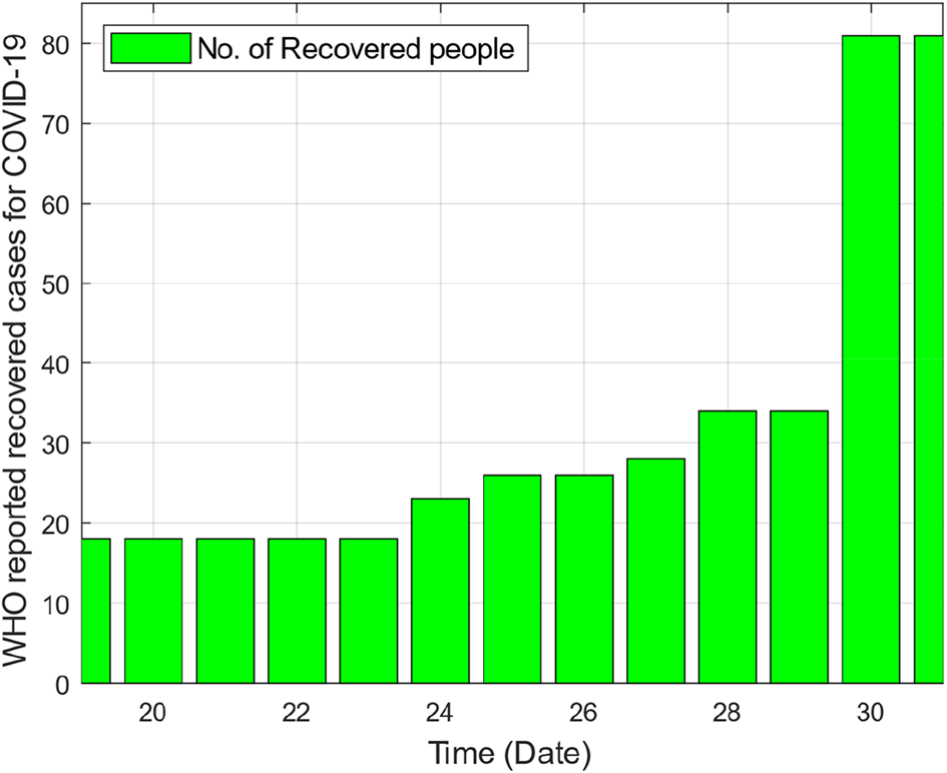
WHO reported recovered cases of COVID-19 from 19th March 2020 till 31st March 2020.

**Figure 5:**
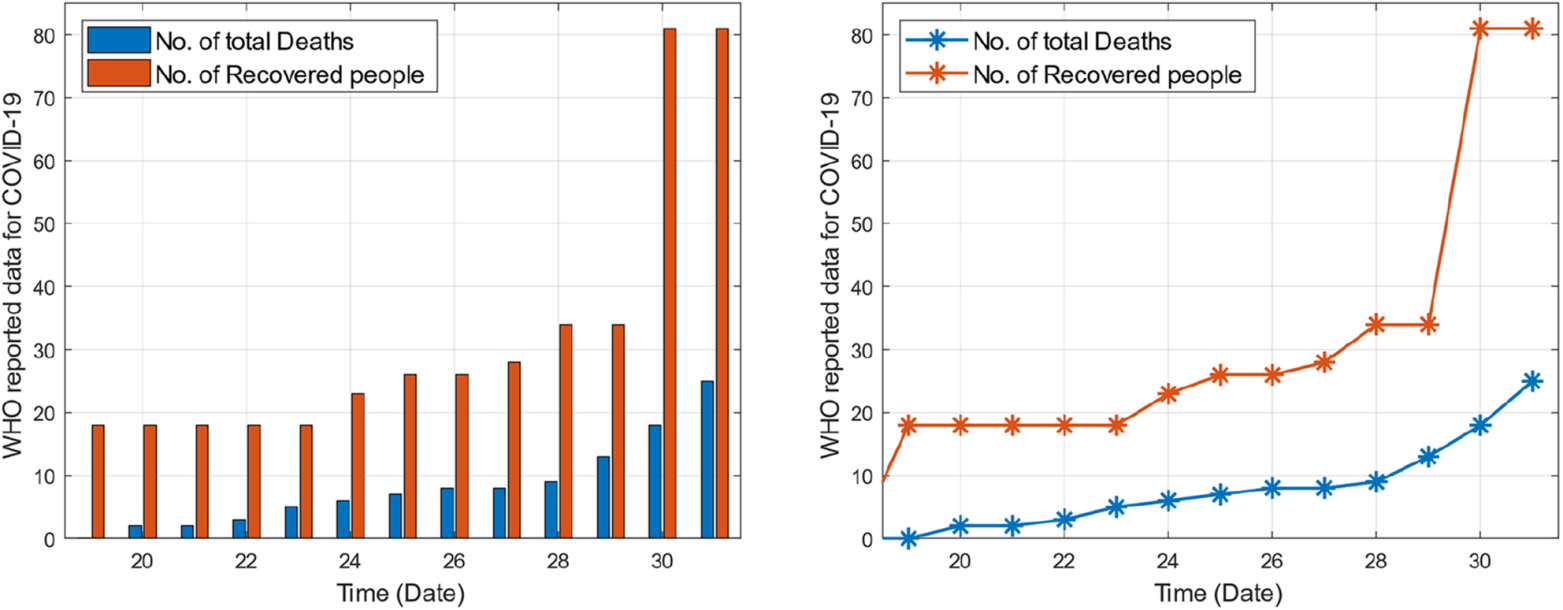
Comparative bar chart and line plot of reported deaths and recovered individuals of COVID-19.

**Figure 6:**
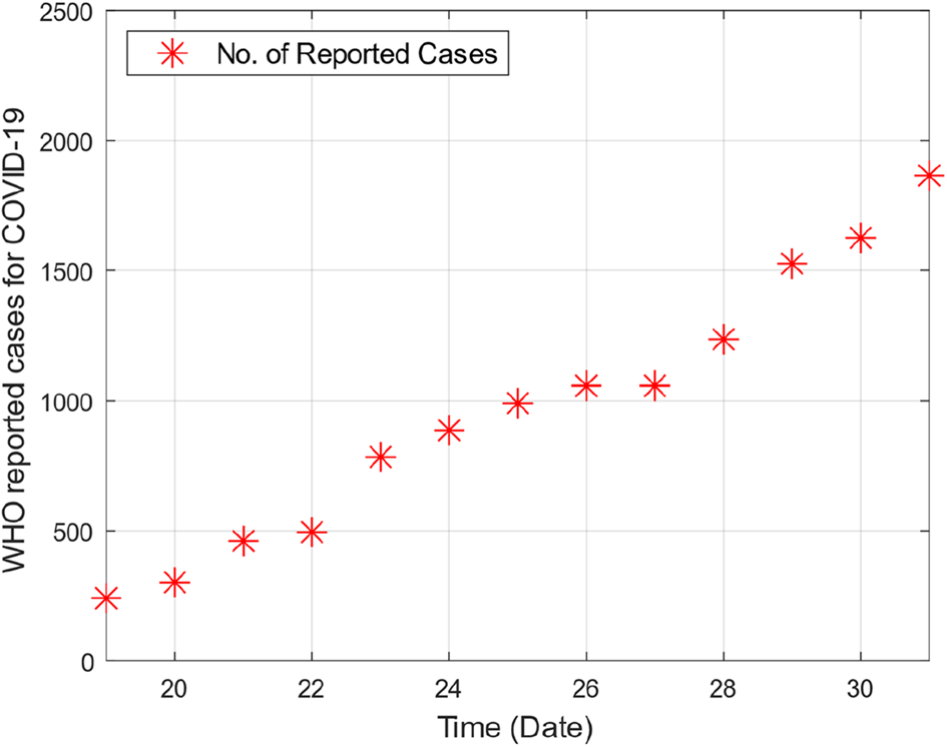
WHO reported cases for COVID-19 from 19th March 2020 till 31st March 2020.

**Figure 7:**
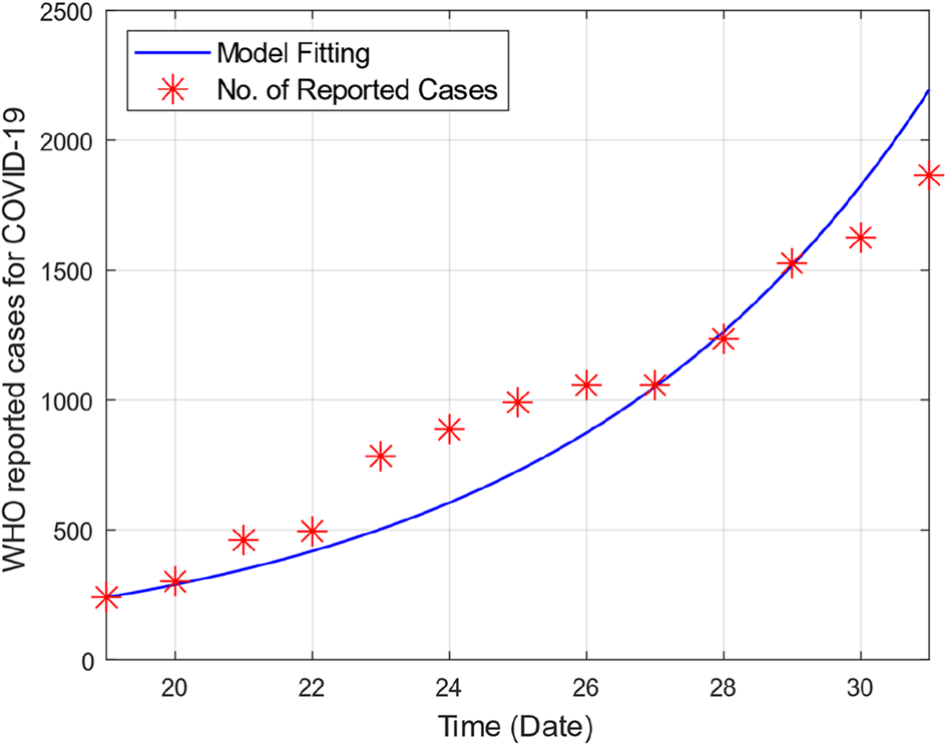
Real data verses model fitting.

This section provides graphical results of the given study. Figure 1 describes the transmission of COVID-19 between different compartments of the total population with the evolution of different parameters which are associated with the dynamics of virus. It is important to study the spread of COVID-19 with the influence of these parameters to predict the spread of this deadly virus. Figure 2 depicts WHO reported cases of infected individuals for COVID-19. Figure 3 displays number of deaths confirmed by WHO due to COVID-19. Figure 4 highlights the total number of recovered individuals of COVID-19 in the selected period of time. Comparative bar chart and line plot of reported deaths and recovered individuals of COVID-19 has been shown in Fig. 5. Figure 6 represents WHO reported cases for COVID-19. Figure 7 shows the comparison between real data from WHO verses present considered model. From the figure, it can be noticed that our model shows a strong agreement with real data collected by WHO. Finally, it is necessary to mention here that from Figs. 2, 3, 4, 5, 6 and 7, all these figures have been plotted for the data collected by WHO from 19th March 2020 till 31st March 2020. From the graphical analysis, one can observe that the rate of spread of COVID-19 in Pakistan in the above mentioned interval is very high as compared to the initial days. Moreover, to obtain the graphical results from the given model we have simulated the given iterative scheme through MATLAB software. Time unit is considered in days for the present model of COVID-19.

**Figure 8:**
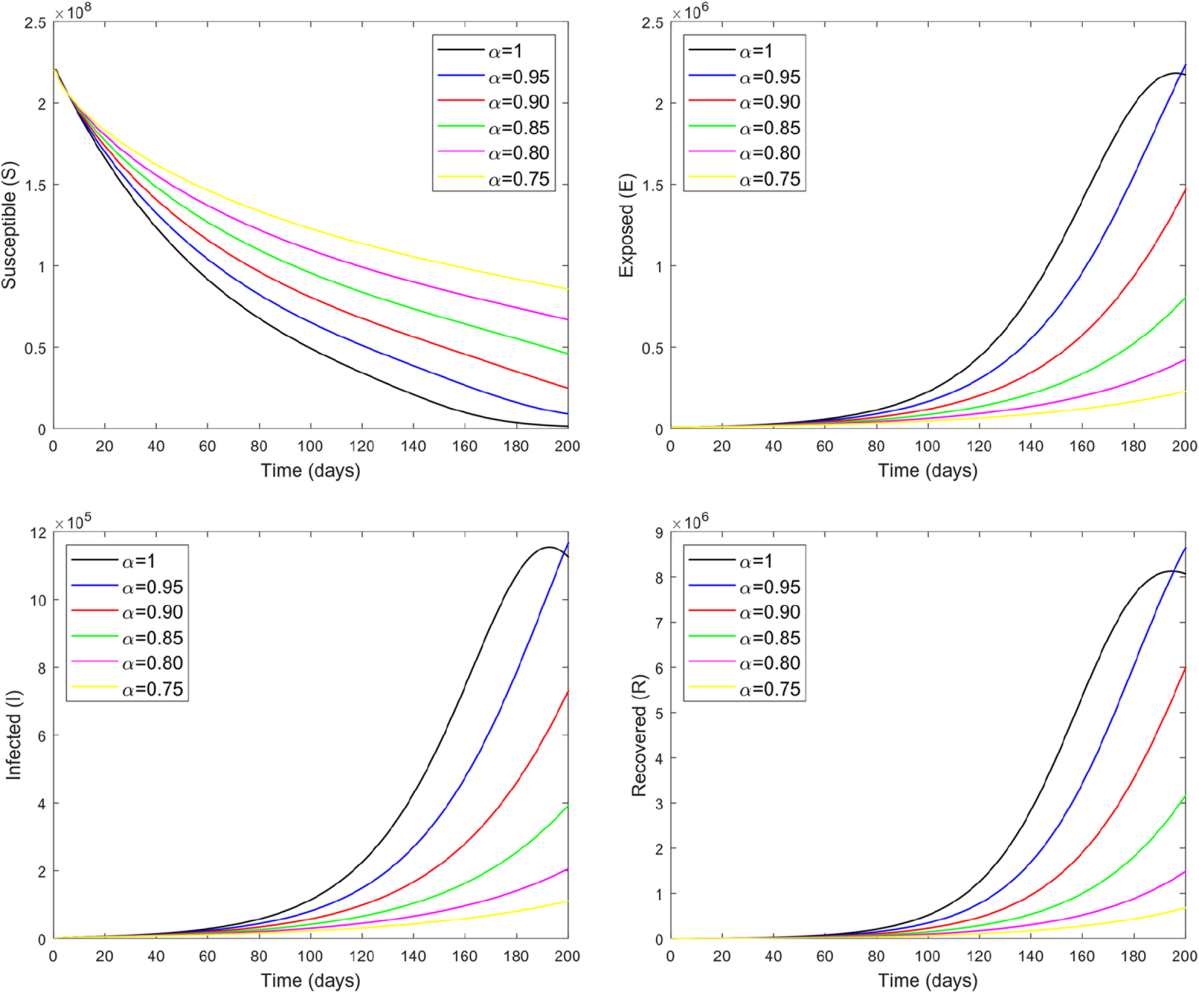
The dynamics of COVID-19 in different classes with different values of fractional parameter $\alpha$.

**Figure 9:**
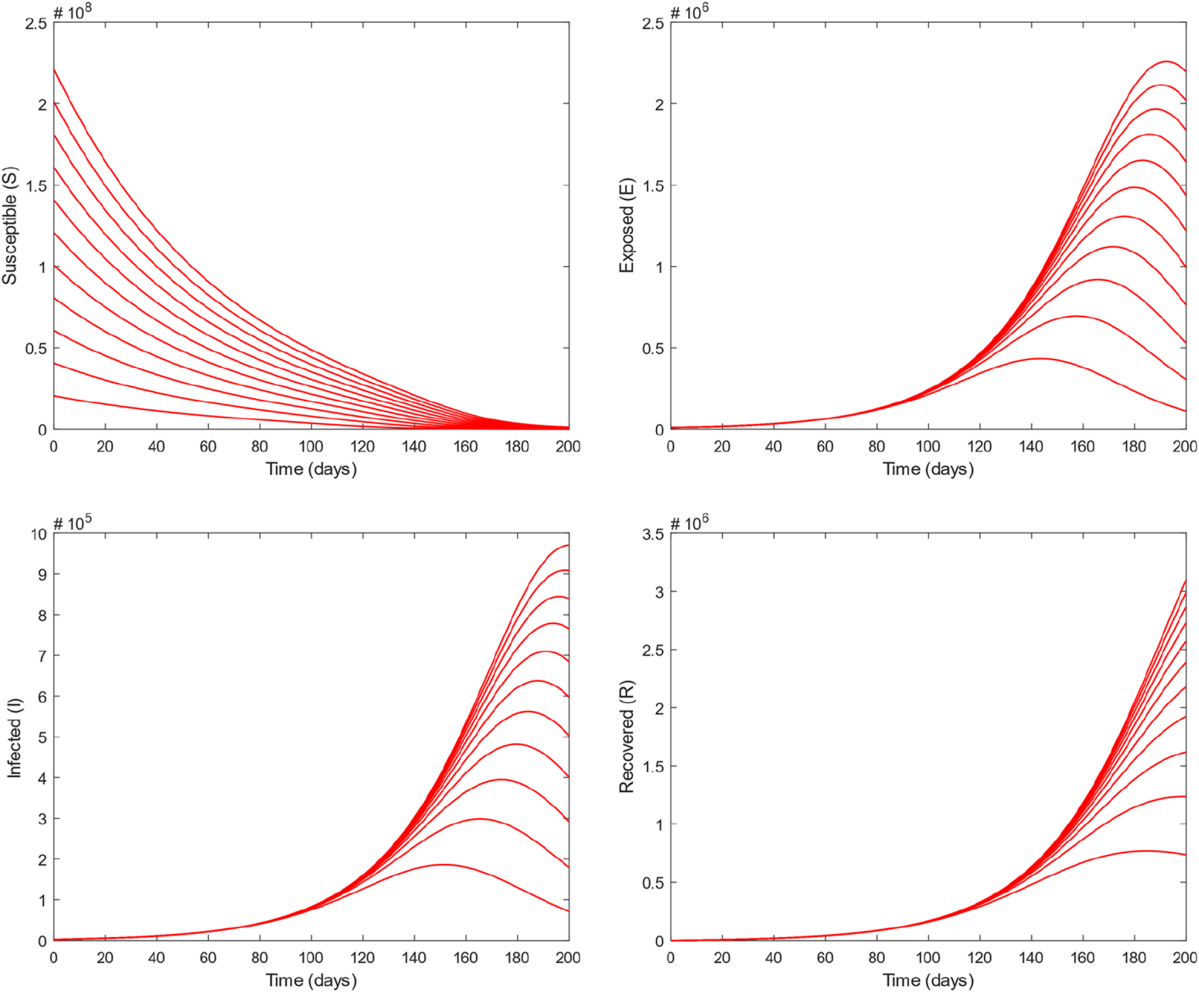
The dynamics of COVID-19 in different classes with different initial values.

Fractional models are important to capture the different memories, decay and crossover behavior. Fractional operator can also demonstrate some upcoming situations for the dynamical models. Figure 8 depicts the influence of different values of fractional parameter $\alpha$ on the dynamics and transmission of COVID-19 for different subclasses of the total population. By varying $\alpha$ while keeping other values fixed, the obtained solutions generates interesting results and gives a variety of solutions of the present model. From the figure, we noticed that by increasing $\alpha$ results to decrease the number of exposed as well as infected individuals and delays the time of reaching the maximum number in each compartment. Decreasing $\alpha$ makes the curves of all population compartments flatter except susceptible class. Furthermore, it can be observed from the figure that we have predicted the dynamics and transmission of COVID-19 for the next 200 days. Finally, Fig. 9 is obtained by considering the different initial values which provides the global asymptotical stability of our model equilibrium.

## Concluding remarks

To understand the spread different infectious diseases including COVID-19 among plants, human or any other animals, mathematicians implement the concept of differentiation and integration to model the dynamics of that disease in the form of ODEs or PDEs that could be used to predict the transmission of that disease within a specific population. For this task, they divide the total population into different compartments. The solution of these systems may deliver an indication to health and other sectors that how severe the spread can be and what parameter is needed to control the spread of virus or disease under consideration. In the present article, we have taken SEIR model for the dynamics and transmission of COVID-19. We have also proved that the given model is bounded and invariant. Two equilibrium points DFE and EE are calculated for the steady state of the presented model. Furthermore, the basic reproduction number ${\mathfrak{R}}_{0}$ is calculated using next generation technique. Through stability analysis, we have concluded that the given model is locally asymptotically stable when ${\mathfrak{R}}_{0} < 1$. It means that by taking ${\mathfrak{R}}_{0} < 1$ the total population tends to DFE and the disease will die out from the population. Additionally, we have generalized the classical SEIR model by applying the recently developed AB fractional model. The real data have been fitted for the classical model i.e. $\alpha = 1$. For the fitted values given in Table 1, we have approximated the basic reproduction number ${\mathfrak{R}}_{0} \approx 2.30748$. A numerical scheme is adopted for the solutions of AB fractional model. After simulation of numerical scheme different graphical results have been obtained. We have considered the unit of time in days. Furthermore, the dynamics and transmission of COVID-19 is predicted for the next 200 days. A decrease is noticed in the infected class by decreasing the values of AB fractional parameter $\alpha$. As the infectious disease in not yet control in Pakistan as well as worldwide. We all know that the researchers and scientist are trying to find proper medical treatment or vaccination to control the spread of COVID-19. In future one may add the quarantine or vaccination or both the classes to the given model and show the effect of these classes on the spread of this deadly infectious disease.

## References

[1] Anderson PK (2004). Emerging infectious diseases of plants: pathogen pollution, climate change and agrotechnology drivers. Trends Ecol. Evol..

[2] Wang D (2020). Clinical characteristics of 138 hospitalized patients with 2019 novel coronavirus-infected pneumonia in Wuhan, China. JAMA.

[3] Sanchez YG, Sabir Z, Guirao JL (2020). Design of a nonlinear SITR fractal model based on the dynamics of a novel coronavirus (COVID). Fractals.

[4] Ñamendys-Silva SA (2020). Respiratory support for patients with COVID-19 infection. Lancet Respir. Med..

[5] World Health Organization. Coronavirus disease 2019 (COVID-19): Situation Report, **73**. https://apps.who.int/iris/handle/10665/331686 (2020).

[6] Zhang Z (2020). A novel COVID-19 mathematical model with fractional derivatives: Singular and nonsingular kernels. Chaos Solitons Fractals.

[7] Shaikh AS, Shaikh IN, Nisar KS (2020). A mathematical model of COVID-19 using fractional derivative: outbreak in India with dynamics of transmission and control. Adv. Differ. Equ..

[8] Baleanu D, Mohammadi H, Rezapour S (2020). A fractional differential equation model for the COVID-19 transmission by using the Caputo–Fabrizio derivative. Adv. Differ. Equ..

[9] Yadav RP, Verma R (2020). A numerical simulation of fractional order mathematical modeling of COVID-19 disease in case of Wuhan China. Chaos Solitons Fractals.

[10] Lu, Z. *et al.* A fractional-order SEIHDR model for COVID-19 with inter-city networked coupling effects. Pre-print at https://arxiv.org/abs/2004.12308 (2020). 10.1007/s11071-020-05848-4 PMC7405792 32836817

[11] Rajagopal K (2020). A fractional-order model for the novel coronavirus (COVID-19) outbreak. Nonlinear Dyn..

[12] Higazy M (2020). Novel fractional order SIDARTHE mathematical model of COVID-19 pandemic. Chaos Solitons Fractals.

[13] Ozarslan R, Ercan A, Bas E (2019). Novel fractional models compatible with real world problems. Fractal Fract..

[14] Qureshi S, Aziz S (2020). Fractional modeling for a chemical kinetic reaction in a batch reactor via nonlocal operator with power law kernel. Phys. A.

[15] Qureshi S, Bonyah E, Shaikh AA (2019). Classical and contemporary fractional operators for modeling diarrhea transmission dynamics under real statistical data. Phys. A.

[16] Bas E, Ozarslan R (2018). Real world applications of fractional models by Atangana–Baleanu fractional derivative. Chaos Solitons Fractals.

[17] Bas E, Acay B, Ozarslan R (2019). Fractional models with singular and non-singular kernels for energy efficient buildings. Chaos.

[18] Evirgen F, Uçar S, Özdemir N (2020). System analysis of HIV infection model with CD4+ T under non-singular kernel derivative. Appl. Math. Nonlinear Sci..

[19] Qureshi S, Yusuf A (2019). Modeling chickenpox disease with fractional derivatives: From caputo to Atangana–Baleanu. Chaos Solitons Fractals.

[20] Prakasha DG, Veeresha P, Baskonus HM (2019). Analysis of the dynamics of hepatitis E virus using the Atangana–Baleanu fractional derivative. Eur. Phys. J. Plus.

[21] Qureshi S, Atangana A (2019). Mathematical analysis of dengue fever outbreak by novel fractional operators with field data. Phys. A.

[22] Koca I (2017). Analysis of rubella disease model with non-local and non-singular fractional derivatives. Int. J. Optim. Control Theor. Appl..

[23] Qureshi S (2020). Monotonically decreasing behavior of measles epidemic well captured by Atangana–Baleanu–Caputo fractional operator under real measles data of Pakistan. Chaos Solitons Fractals.

[24] Sweilam NH, Al-Mekhlafi SM, Baleanu D (2019). Optimal control for a fractional tuberculosis infection model including the impact of diabetes and resistant strains. J. Adv. Res..

[25] Gao W, Veeresha P, Prakasha DG, Baskonus HM (2020). Novel dynamic structures of 2019-nCoV with nonlocal operator via powerful computational technique. Biology.

[26] Atangana, A., & Araz, S. I. Mathematical model of COVID-19 spread in Turkey and South Africa: Theory, methods and applications. Pre-print at 10.1101/2020.05.08.20095588 (2020). 10.1186/s13662-020-03095-w PMC7758164 33391372

[27] Mohammad M, Trounev A (2020). On the dynamical modeling of COVID-19 involving Atangana–Baleanu fractional derivative and based on Daubechies framelet simulations. Chaos Solitons Fractals.

[28] Arif M, Ali F, Sheikh NA, Khan I, Nisar KS (2019). Fractional model of couple stress fluid for generalized Couette flow: A comparative analysis of Atangana–Baleanu and Caputo–Fabrizio fractional derivatives. IEEE Access.

[29] Arif M, Ali F, Khan I, Nisar KS (2020). A time fractional model with non-singular kernel the generalized Couette flow of couple stress nanofluid. IEEE Access.

[30] Sheikh NA, Ali F, Khan I, Gohar M, Saqib M (2017). On the applications of nanofluids to enhance the performance of solar collectors: A comparative analysis of Atangana–Baleanu and Caputo–Fabrizio fractional models. Eur. Phys. J. Plus.

[31] Doungmo Goufo EF (2016). Chaotic processes using the two-parameter derivative with non-singular and non-local kernel: Basic theory and applications. Chaos.

[32] Gómez-Aguilar JF, Atangana A, Morales-Delgado VF (2017). Electrical circuits RC, LC, and RL described by Atangana–Baleanu fractional derivatives. Int. J. Circuit Theory Appl..

[33] Van den Driessche P, Watmough J (2002). Reproduction numbers and sub-threshold endemic equilibria for compartmental models of disease transmission. Math. Biosci..

[34] Atangana, A., & Baleanu, D. New fractional derivatives with nonlocal and non-singular kernel: theory and application to heat transfer model. *arXiv preprint* arXiv:1602.03408 (2016).

[35] Sene N (2020). SIR epidemic model with Mittag–Leffler fractional derivative. Chaos Solitons Fractals.

[36] Toufik M, Atangana A (2017). New numerical approximation of fractional derivative with non-local and non-singular kernel: application to chaotic models. Eur. Phys. J. Plus.

[37] Population of Pakistan. https://www.worldometers.info/world-population/pakistan-population.

[38] Life expectancy in Pakistan. https://www.worldlifeexpectancy.com/pakistan-life-expectancy.

